# Total Abstinence and Common Sense

**Published:** 1886-12-04

**Authors:** 


### TOTAL ABSTINENCE AND COMMON SENSE.

Common sense was the characteristic of the utterances of the late Bishop of Manchester, and his successor, Dr. Moorhouse, has inherited the same quality. Speaking on the 24th inst. he is reported to have said:

"Many years ago I signed a conditional pledge. I went on very well until towards the close of the second year, and then I broke down utterly. My doctor told me I must either give up half my work or take some light stimulants with my principal meals. It was ridiculous to think of giving up half my work, therefore I tried the stimulants, and I never had the symptoms again. I suppose I am one of the thousands who have not the power of easily digesting great masses of food, and cannot engage in active mental labour without nervous excitement. Thousands of men doing the greater part of the intellectual work of England belong to the same class, and it would be monstrous for these men either to commit suicide or give up half their work, so they are bound to take the stimulus. I, however, observe a rigid rule—never to drink alcoholic liquor except at meal times."

These are words of common sense based upon experience. It cannot, perhaps, be denied that everyone in robust health would do as well, or better, without alcohol than with it. There is also evidence that under conditions of exposure to great heat and great cold, to extreme hardships and exposure, those who have not taken alcohol have come off much better than those who have. The use of alcohol, then, is not usually necessary to a healthy person under 40 years of age. He leads the most healthy life who lives temperately and soberly in all things, avoiding all excess. But all men are not healthy; many live in these days at too great a rate, they work too hard, they lead lives of great excitement, and somehow or other, as in the Bishop's case, they find they must take a certain amount of alcohol to keep them in good health. Furthermore, it is alcohol and this *alone* that seems to supply them with what they need, and if its use is given up from conscientious motives their health suffers. Much must in all these cases be allowed for the idiosyncrasies of the individual. To old people, for instance, wine is usually a great comfort. The wearinesses and annoyances of age call for a slight stimulant and narcotic, and the digestion needs strengthening. The failure in force is well met by a substance whose distribution in the system yields much power without effort. It is, in short, wise to have no hobbies on the subject, but to trust to and be guided by one's medical attendant, and one's own sober sense and experience.

Dr. Monsey, a clever wag, was once travelling with a friend in Wales, and pointing out to him the features of the country they passed through. "That moderately-sized eminence, sir," he said, "is locally known as Black Mountain." "Any story connected with it?" queried the friend. "Yes," responded the humorous doctor; "once a couple of lovers went up it from this village, and never returned." "Indeed," said the friend, with an apprehensive glance, "what became of them?" "Oh, they descended the other side," said Monsey.

